# Clinical characteristics, autoantibody profiles, and therapeutic outcomes of juvenile systemic sclerosis: an 11-year single-center experience from China

**DOI:** 10.3389/fimmu.2026.1818010

**Published:** 2026-05-15

**Authors:** Yating Wang, Wenjing Tang, Yu Zhang, Zhiyong Zhang, Yunfei An, Xuemei Tang

**Affiliations:** 1 Department of Rheumatology and Immunology Children’s Hospital of Chongqing Medical University, National Clinical Research Center for Children and Adolescents’ Health and Diseases, Ministry of Education Key Laboratory of Child Development and Disorders, Chongqing, China; 2 Chongqing Key Laboratory of Child Rare Diseases in Infection and Immunity, Chongqing, China

**Keywords:** autoantibodies, disease activity, juvenile systemic sclerosis, modified Rodnan skin score, pediatric rheumatology, tocilizumab, treatment outcome

## Abstract

**Objective:**

Juvenile systemic sclerosis (jSSc) is a rare but serious rheumatic disease in children that significantly impacts growth and development. This study aimed to summarize the clinical characteristics and disease patterns of jSSc and to evaluate the treatment response over an 11-year period at our center.

**Methods:**

Twenty-one pediatric jSSc patients treated at our center from January 2015 to December 2025 were retrospectively analyzed. Classification was based on the 2013 American College of Rheumatology (ACR)/European Alliance of Associations for Rheumatology (EULAR) systemic sclerosis classification criteria. Overall disease severity and multiorgan activity were assessed using the Juvenile Systemic Sclerosis Severity Score (J4s; range 0–40), while skin involvement was specifically evaluated using the modified Rodnan skin score (mRSS; range 0–51). The median follow-up duration was 2.3 years (range 0.5–7.2 years). Paired t-tests and Wilcoxon signed-rank tests were used to compare pre- and posttreatment parameters, with p < 0.05 considered statistically significant.

**Results:**

Twenty-one patients were enrolled. Skin sclerosis was the most common symptom (100.0%). Antinuclear antibody (ANA) was positive in 16 cases (76.2%), with anti-single-stranded DNA (anti-ssDNA) antibody being the most common specific antibody (23.8%), followed by anti-PM-Scl antibody (19.0%). Glucocorticoids and methotrexate were commonly used as initial therapies. After a median treatment duration of 24 months, overall disease severity (J4s) significantly decreased (5.94 ± 1.66 vs 2.67 ± 2.22, p = 0.003), and skin involvement (mRSS) also improved significantly (10.0 ± 9.21 vs 7.42 ± 8.26, p = 0.008). Fourteen patients received biologic agents, with 11 using tocilizumab. During the follow-up, no deaths or serious adverse events occurred.

**Conclusions:**

jSSc is a rare and severe multiorgan disease with unique autoantibody profiles in Chinese children. The J4s and mRSS serve as objective assessment tools for evaluating overall disease severity and skin involvement, respectively, and are effective in monitoring treatment response. Early diagnosis and individualized therapy, including the adjunctive use of tocilizumab, may improve clinical outcomes.

## Introduction

Systemic sclerosis (SSc) is a rare rheumatic disease characterized by microvascular damage, immune system dysregulation, and progressive fibrosis of the skin and internal organs. When the disease occurs before the age of 18, it is termed juvenile systemic sclerosis (jSSc) (1). Compared with adult SSc, jSSc differs in clinical presentation, disease course, and prognosis, but precise epidemiological data remain limited globally (2). A study based on the UK and Irish populations reported an annual incidence of approximately 0.202 cases per million children, highlighting its rarity (3). However, epidemiological data exhibit significant ethnic and geographic variability. In the United States, African American children demonstrate a higher prevalence and a more severe disease course than Caucasian children (4). In Asia, recent nationwide surveys from Japan and Korea suggest a lower incidence (approximately 0.05–0.1 cases per million) but a higher proportion of the diffuse cutaneous subtype compared to European cohorts, indicating potential genetic and environmental influences on disease expression (5, 6).

jSSc poses serious threats to children’s growth, development, and long-term quality of life (1, 7, 8). The clinical spectrum ranges from early manifestations, such as Raynaud’s phenomenon and skin sclerosis, to late-stage complications, including interstitial lung disease (ILD), pulmonary arterial hypertension (PAH), cardiac involvement, and gastrointestinal complications, which can lead to irreversible organ damage or death. Therefore, early recognition, assessment of disease activity, and intervention are crucial for improving prognosis (9). The diagnosis of jSSc primarily follows the 2013 American College of Rheumatology (ACR)/European Alliance of Associations for Rheumatology (EULAR) SSc classification criteria, which incorporate multiple indicators, including skin involvement extent, nailfold capillary abnormalities, specific autoantibodies, and visceral organ manifestations, thereby improving diagnostic sensitivity, especially for early and atypical cases (10, 11). Based on the extent of skin involvement, jSSc can be classified into diffuse cutaneous, limited cutaneous, and sine scleroderma subtypes. Overlap syndromes with other rheumatic diseases are also relatively common in pediatric patients (12). Due to its rarity, diverse clinical presentations, and lack of specific diagnostic markers, jSSc often experiences diagnostic delays. Available global data indicate that the median diagnostic delay ranges from 1.0 to 2.8 years (1). Specifically, European registries (such as the SHARE initiative) report a median delay of approximately 1.9 years, while studies in Asian populations have observed delays of up to 2.3 years, often attributed to the late recognition of Raynaud’s phenomenon as a systemic harbinger in pediatric patients (7, 13).

Current jSSc management strategies are primarily based on adult evidence, expert consensus (such as the European SHARE initiative), and small-scale retrospective studies due to the lack of prospective randomized controlled trials in children (14). The 2024 Hamburg consensus conference updated the jSSc treatment guidelines, emphasizing the importance of treat-to-target strategies (15). Glucocorticoids (GCs) and methotrexate (MTX) are foundational drugs for controlling disease activity, while biologic agents such as the IL-6 receptor inhibitor tocilizumab and B-cell depletion agent rituximab are increasingly used for refractory cases or severe visceral involvement (such as progressive ILD), demonstrating preliminary efficacy and safety (9, 10). Novel targeted therapies such as JAK inhibitors have also been successfully applied in individual cases, although the evidence level remains extremely low (16).

Clinical research on jSSc is relatively limited. Whether the clinical phenotypes, autoantibody profiles, and treatment responses differ from those of other populations remains unclear. The primary aim of this study was to systematically characterize the clinical phenotypes and autoantibody profiles of jSSc within a Chinese cohort over an 11-year period. As secondary outcomes, we aimed to evaluate disease activity and severity using standardized tools [Juvenile Systemic Sclerosis Severity Score (J4s) and modified Rodnan skin score (mRSS)] and to preliminarily explore the efficacy and safety of tocilizumab in real-world clinical practice.

## Methods

### Study subjects

Pediatric jSSc patients evaluated and treated at our center from January 2015 to December 2025 were retrospectively reviewed for inclusion in this study. Inclusion criteria were as follows: 1) age ≤18 years, 2) met the 2013 ACR/EULAR systemic sclerosis classification criteria (10), 3) a minimum follow-up duration of 6 months, and 4) complete medical records. Exclusion criteria were as follows: 1) other types of scleroderma (such as localized scleroderma and scleroderma-like diseases) and 2) coexisting severe infection, malignancy, or other serious life-threatening diseases present simultaneously at the time of initial jSSc diagnosis (baseline). It should be noted that infections occurring during the follow-up period were not criteria for exclusion but were instead strictly monitored and recorded as treatment-related adverse events.

### Classification criteria

The 2013 ACR/EULAR systemic sclerosis classification criteria were used (10). These criteria stipulate that if proximal skin sclerosis (thickening of skin on both hands extending proximal to the metacarpophalangeal joints) is present, this single criterion suffices for classification. If proximal skin sclerosis is absent, a cumulative score ≥9 points from the following seven-item scoring system is needed: finger skin thickening [2 points for puffy fingers/4 points for sclerodactyly distal to metacarpophalangeal (MCP) joints], fingertip lesions (2 points for fingertip ulcers/3 points for fingertip pitting scars), telangiectasia (2 points), nailfold capillary abnormalities (2 points), pulmonary arterial hypertension and/or interstitial lung disease (2 points), Raynaud’s phenomenon (3 points), and SSc-related antibody positivity (3 points).

### Data collection and definitions

Through the electronic medical record system, two researchers independently collected and verified the following data. 1) Demographics and basic disease characteristics: sex, age at onset, age at diagnosis, and diagnostic delay time (from first symptom to confirmed diagnosis). Age at onset was defined as the age at which the first non-Raynaud’s symptom (e.g., skin hardening, joint swelling, or finger puffiness) attributable to jSSc appeared. Skin subtypes were classified based on the maximum extent of involvement observed throughout the entire disease course as diffuse cutaneous SSc (dcSSc; skin thickening extending beyond elbows/knees, often involving trunk) and limited cutaneous SSc (lcSSc; skin thickening limited to distal to elbows/knees). 2) Clinical manifestations: Skin/vascular involvement, including the extent of skin thickening, mRSS (range 0–51), fingertip ulcers, and Raynaud’s phenomenon [defined as typical triphasic (pallor–cyanosis–rubor) or biphasic (cyanosis–rubor) color changes at finger/toe tips during cold exposure or emotional stress]. Joint and muscle involvement included arthritis (joint swelling or tenderness confirmed by examination), tendon friction rubs, and myositis. Visceral organ involvement was systematically evaluated using standardized objective examinations. Specifically, ILD was assessed using high-resolution computed tomography (HRCT). PAH was systematically screened via transthoracic echocardiography in all patients, and suspected cases were defined and evaluated according to the 2022 European Society of Cardiology/European Respiratory Society (ESC/ERS) ESC/ERS guidelines, with right heart catheterization (RHC) utilized for definitive diagnosis when clinically indicated (17). Furthermore, isolated diffusing capacity for carbon monoxide (DLCO) impairment was strictly defined as a DLCO < 80% of the predicted value, but only in the absence of both HRCT-confirmed structural lung disease and echocardiography/RHC-confirmed PAH (18). Cardiac involvement was screened via comprehensive echocardiography in all patients, with cardiac magnetic resonance (CMR) performed when specific myocardial or pericardial involvement was suspected. Gastrointestinal involvement and renal involvement were confirmed via appropriate endoscopy, imaging, or laboratory parameters. 3) Disease activity and severity assessment: mRSS scores at diagnosis or before treatment were collected. The J4s (range 0–40) was used to assess disease activity and overall disease severity, with higher scores indicating more severe multiorgan involvement (3). 4) Laboratory tests: Antinuclear antibody (ANA; by indirect immunofluorescence, titer ≥ 1:80 considered positive) and specific autoantibody profiles (by immunoblot or ELISA, including anti-Scl-70, anti-centromere, anti-PM-Scl, anti-U1-RNP, anti-Ro-52, and anti-RNA polymerase III) were collected for all patients. 5) Treatment regimens: Detailed records of systemic drug therapies, including GCs, conventional synthetic disease-modifying antirheumatic drugs (DMARDs), biologic agents, and targeted synthetic DMARDs, were maintained. Specifically, the standardized regimen for tocilizumab consisted of an intravenous (IV) infusion administered once monthly. The dosage was weight-based: 12 mg/kg for patients weighing <30 kg and 8 mg/kg for patients weighing ≥30 kg. Additionally, hepatoprotective drugs used in this cohort primarily included glutathione and glycyrrhizic acid preparations administered to manage transient transaminase elevations. 6) Follow-up and outcomes: The last follow-up time was recorded, and the total follow-up duration (from diagnosis to last follow-up or data cutoff date) was calculated. A minimum follow-up duration of 6 months was required for inclusion in this study. The “posttreatment” assessment for disease activity scores (J4s and mRSS) was strictly defined as the evaluation performed at the final follow-up visit for each patient to assess the maximal therapeutic response. Posttreatment changes in the mRSS and J4s scores, imaging, and pulmonary function improvements, as well as adverse events, were meticulously documented. For patients evaluating pulmonary involvement, chest HRCT was routinely repeated every 6 months to monitor structural lung changes. It should be noted that while severe infection or malignancy at baseline were criteria for exclusion, any such conditions occurring during the follow-up period were not grounds for exclusion but were instead recorded as treatment-related adverse events. Serious adverse events (SAEs) were defined in accordance with the International Council for Harmonisation (ICH) E6 (R2) Guideline for Good Clinical Practice [ICH E6 (R2)] (19) as any medical occurrence that resulted in death, was life-threatening, required inpatient hospitalization or prolongation of existing hospitalization, or resulted in persistent or significant disability or incapacity.

### Assessment methods

The mRSS assessed skin thickening in 17 body areas, each scored 0–3 points, with a total score of 0–51 points, with higher scores indicating more severe skin involvement (9). The J4s evaluated nine domains—general, vascular, skin, osteoarticular, muscle, gastrointestinal, respiratory, cardiac, and renal—with a total score of 0–40 points, with higher scores indicating more severe disease (11). Pulmonary function tests included forced vital capacity (FVC) and diffusing capacity for DLCO.

### Statistical methods

Continuous variables are expressed as medians (ranges) or means ± standard deviations, and categorical variables are expressed as numbers (percentages). Pre- and posttreatment J4 comparisons used paired t-tests, and mRSS comparisons used Wilcoxon signed-rank tests. p < 0.05 was considered statistically significant. Statistical analyses were performed using the SPSS 26.0 software.

## Results

### Demographics

A total of 21 pediatric jSSc patients met the inclusion criteria and were enrolled in this study. Among them, six were male and 15 female, with a female-to-male ratio of 2.5:1. The median age at diagnosis was 8 years (range 5–14), the median age at onset was 6 years (range 2–13), and the median diagnostic delay time was 1 year (range 0–12). Based on the extent of skin involvement, 14 cases were of the diffuse type, and seven were of the limited type. The median follow-up time was 2.3 years (range 0.5–7.2 years).

### Clinical manifestations

All patients had varying degrees of skin involvement. At the time of initial diagnosis, proximal skin sclerosis (skin thickening extending proximal to the metacarpophalangeal joints) was present in eight cases (38.1%), while fingertip ulcers were observed in three cases. However, during the disease course and follow-up, skin involvement progressed in several patients, resulting in a total of 14 patients (66.7%) ultimately exhibiting skin thickening that extended proximally to the elbows or knees, thereby being classified as dcSSc. Raynaud’s phenomenon occurred in four cases (19.0%), manifesting as pallor, cyanosis, and rubor of extremities during cold exposure or emotional excitement. Skin biopsies were performed in nine cases, all showing pathological changes: four were diagnosed with “scleroderma-like features” (subtype unspecified), and five showed non-specific organic microangiopathy. According to the Couto classification, two were in the early stage, three were in the active stage, and four were in the late stage. The mRSS ranged from 2 to 33 points, with a mean of 11 ± 9.53 points.

Despite the relatively low prevalence of Raynaud’s phenomenon (19.0%), all 17 patients without this feature met diagnostic criteria through alternative pathways: eight (47.1%) via the major criterion of proximal skin sclerosis alone at baseline and nine (52.9%) via cumulative scoring (≥9 points), primarily driven by finger skin thickening, nailfold capillary abnormalities, ANA positivity, and pulmonary involvement. This indicates that even in the absence of Raynaud’s phenomenon, the combination of other clinical features and laboratory findings can still meet diagnostic criteria.

Joint involvement occurred in 12 cases, all during the early disease stage, with no new cases during follow-up, mainly manifesting as joint pain and limited mobility, with one case accompanied by tendon contracture. Muscle involvement occurred in two cases, with one case showing muscle atrophy. Gastrointestinal involvement occurred in five cases, including gastritis in four cases (all confirmed by endoscopy), reflux esophagitis in one case, and persistent diarrhea at diagnosis that progressed to intestinal obstruction during follow-up in one case.

Pulmonary involvement occurred in a total of six cases (28.6%). This included structural ILD confirmed by HRCT in three cases (one of whom concurrently developed pulmonary arterial hypertension). Notably, the remaining three cases exhibited isolated DLCO impairment (DLCO < 80% predicted). All patients with isolated DLCO reduction underwent HRCT and comprehensive echocardiography, which were completely negative, thereby explicitly ruling out structural lung fibrosis and PAH. These patients were strictly classified as having isolated DLCO impairment and were not overclassified as having ILD. All patients underwent echocardiography, with only one case suggesting cardiac involvement (specifically, a mild reduction in ventricular diastolic function) and no cases of myocarditis or pericarditis. No renal crises occurred in the entire cohort. The J4s ranged from 4 to 10 points, with a mean of 5.8 ± 1.62 points (**Table 1**).

**Table 1:** Demographic and clinical characteristics of the cohort (n = 21).

Characteristics	Value
Demographic characteristics	
Sex, n (%)	
Female	15 (71.4)
Male	6 (28.6)
F:M ratio	2.5:1
Age at diagnosis (years), median (range)	8 (5–14)
Age at onset (years)*, median (range)	6 (2–13)
Diagnostic delay (years), median (range)	1 (0–12)
Clinical subtype (cumulative maximum), n (%)**	
Limited cutaneous	7 (33.3)
Diffuse cutaneous	14 (66.7)
Median follow-up (years), median (range)	2.3 (0.5–7.2)
Clinical manifestations	Patients, n (%)
Skin involvement	21 (100.0)
Raynaud’s phenomenon	4 (19.0)
Skin sclerosis	18 (85.7)
Proximal skin sclerosis (at initial diagnosis)***	8 (38.1)
Fingertip ulcers	3 (14.3)
Joint involvement	12 (57.1)
Arthritis	8 (38.1)
Tendon contracture	1 (4.8)
Muscle involvement	2 (9.5)
Gastrointestinal involvement	5 (23.8)
Gastritis	4 (19.0)
Reflux esophagitis	1 (4.8)
Diarrhea	1 (4.8)
Intestinal obstruction	1 (4.8)
Pulmonary involvement	6 (28.6)
Interstitial lung disease	3 (14.3)
DLCO impairment	3 (14.3)
Pulmonary arterial hypertension	1 (4.8)
Cardiac involvement	1 (4.8)
Renal crisis	0 (0.0)

Continuous data are presented as the median (range). Categorical data are presented as numbers (percentages).

DLCO, diffusing capacity for carbon monoxide.

* Age at onset was defined as the appearance of the first non-Raynaud’s symptom attributable to jSSc.

** Classified based on the maximum extent of skin involvement observed throughout the entire disease course and follow-up period, reflecting disease progression.

*** Refers specifically to skin thickening extending proximal to the metacarpophalangeal joints at the time of initial baseline diagnosis, serving as the major American College of Rheumatology (ACR)/European Alliance of Associations for Rheumatology (EULAR) classification criterion.

### Autoantibodies

Twenty-one patients underwent autoantibody testing. ANA was positive (titer ≥ 1:80) in 16 cases (76.2%). The distribution of autoantibody profiles was as follows: anti-single-stranded DNA (anti-ssDNA) antibody positive in five cases (23.8%), anti-PM-Scl antibody positive in four cases (19.0%), and anti-Scl-70 antibody positive in only two cases (9.5%). Other positive antibodies included anti-proliferating cell nuclear antigen (anti-PCNA) antibody, anti-Ro-52 antibody, anti-histone antibody, and anti-centromere antibody positive in two cases (9.5%) each, and anti-nuclear ribonucleoprotein (anti-nRNP) antibody positive in one case (4.8%). Five patients (23.8%) were all negative (**Table 2**).

**Table 2:** Autoantibody profile of the cohort (n = 21).

Autoantibody	Number of patients, n (%)
ANA positive	16 (76.2)
Anti-ssDNA positive	5 (23.8)
Anti-PM-Scl positive	4 (19.0)
Anti-Scl-70 positive	2 (9.5)
Anti-PCNA positive	2 (9.5)
Anti-Ro-52 positive	2 (9.5)
Anti-histone positive	2 (9.5)
Anti-centromere positive	2 (9.5)
Anti-nRNP positive	1 (4.8)
All negative	5 (23.8)

ANA, antinuclear antibody; ssDNA, single-stranded DNA; PCNA, proliferating cell nuclear antigen; nRNP, nuclear ribonucleoprotein.

### Treatment

Most patients received immunosuppressive therapy. Except for three cases, 18 patients (85.7%) used GCs. Methotrexate was the most commonly used (13, 61.9%) DMARD in this cohort, followed by mycophenolate mofetil (7, 33.3%). Fourteen patients (66.7%) received biologic agents, including tocilizumab in 11 patients and rituximab, infliximab, and etanercept in one patient each. Nine cases (42.9%) used a combination regimen of “1 DMARD + 1 biologic agent”. Other treatment modalities included intravenous immunoglobulin (IVIG) and topical tacrolimus (**Table 3**).

**Table 3:** Treatment modalities administered (n = 21).

Therapeutic strategy	Patients, n (%)
GCs	18 (85.7)
DMARDs	17 (81.0)
Methotrexate	13 (61.9)
Mycophenolate mofetil	7 (33.3)
Cyclophosphamide	1 (4.8)
Biologic agents	14 (66.7)
Tocilizumab	11 (52.4)
Rituximab	1 (4.8)
Infliximab	1 (4.8)
Etanercept	1 (4.8)
JAK inhibitors	2* (9.5)
Tofacitinib	2
Upadacitinib	1
Hydroxychloroquine	3 (14.3)
Intravenous immunoglobulin (IVIG)	1 (4.8)
Topical immunosuppressants	3 (14.3)
UVA phototherapy	2 (9.5)
Tendon contracture release	1 (4.8)

DMARDs, disease-modifying antirheumatic drugs; GCs, glucocorticoids; JAK, Janus kinase; IVIG, intravenous immunoglobulin; UVA, ultraviolet A.

*Two patients received JAK inhibitors, one of whom received both tofacitinib and upadacitinib sequentially.

### Disease activity evaluation

Statistical analysis of pre- and posttreatment showed significant improvements in both the J4s and mRSS scores (**Table 4**). The posttreatment scores were measured at a median interval of 24 months (range 6–86 months) after the initiation of therapy. Initial clinical improvements, reflected by a discernible reduction in the J4s and mRSS, were typically observed between 3 and 6 months following the introduction of systemic immunosuppressive treatment. During follow-up, no deaths, renal crises, or serious adverse reactions occurred. Two patients developed mild Cushing syndrome after GC use, which resolved after dose reduction; one patient developed mild liver function abnormalities after methotrexate use, which normalized after adding hepatoprotective drugs. No serious infections, allergic reactions, or other adverse reactions were observed in patients receiving biologic agents.

**Table 4:** Comparison of disease activity indicators before and after treatment.

Efficacy indicator	Pretreatment (mean ± SD)	Posttreatment* (mean ± SD)	Mean reduction (%)	P-value
J4s score (n = 18)**	5.94 ± 1.66	2.67 ± 2.22	55.1	0.003
mRSS (n = 19)***	10.0 ± 9.21	7.42 ± 8.26	25.8	0.008

J4s comparison by paired t-test; mRSS comparison by Wilcoxon signed-rank test.

J4s, Juvenile Systemic Sclerosis Severity Score; mRSS, modified Rodnan skin score; SD, standard deviation.

* Posttreatment scores were recorded at the final follow-up, with a median treatment duration of 24 months (range 6–86 months). Clinical improvements were generally first noted within 3–6 months of treatment initiation.

** Posttreatment J4s data were missing for 3 patients.

*** Posttreatment mRSS data were missing for 2 patients.

### Tocilizumab treatment analysis

Pre- and posttreatment analyses were performed for patients receiving tocilizumab in the cohort. Among 11 patients, except for one patient who discontinued treatment due to leukopenia after tocilizumab use, all other patients tolerated tocilizumab well. All patients received baseline immunosuppressive therapy (GCs combined with DMARDs). The median time from jSSc diagnosis to tocilizumab initiation was 12 months (range 3–48 months), and the median time from the introduction of initial GCs/DMARDs to tocilizumab initiation was 10 months (range 2–46 months). The median follow-up duration on tocilizumab therapy for the 11 patients was 16 months (range 3–30 months). One patient with abnormal chest CT before treatment showed complete normalization of chest imaging after treatment, with no patients showing deterioration of pulmonary imaging. Some patients showed improvements in FVC and/or DLCO. Most patients showed improvements in the mRSS and J4s compared to pretreatment (**Table 5**).

**Table 5:** Clinical outcomes in patients treated with tocilizumab.

Pt	Concomitant therapy	Number of TCZ courses	Follow-up duration on TCZ (months)	CT change	DLCO (% pred)	FVC (% pred)	mRSS (pre/post)	J4s (pre/post)
1	GC+MTX	10	10	+ → +	53/67	82/97	32/20	8/2
2	GC+MMF	27	27	+ → +	71/71	75/93	4/2	5/2
3	GC+MTX	9	9	− → −	81/85	94/96	4/2	4/1
4	GC+MTX	16	16	− → −	94/95	113/112	12/6	7/5
5	GC+MMF	30	30	+ → −	80/89	90/92	4/1	5/1
6	GC+MTX	16	16	− → −	76/83	91/97	2/2	4/1
7	GC+MTX	29	29	− → −	NA	98	6/4	6/2
8	GC+MMF	12	12	− → −	83/74	96/105	4/4	5/3
9	GC+MTX	20	20	− → −	NA	104/109	12/10	4/1
10	GC+MTX	3	3	− → −	NA	98	6/4	5/4
11*	GC+CYC	6	6	− → −	103/NA	92/93	9/9	7/5
Pooled (n = 10)	-–	16 (3–30)	16 (3–30)	-–	-–	-–	9.6 ± 8.7/5.4 ± 5.5	5.7 ± 1.4/2.5 ± 1.5

TCZ was administered via intravenous (IV) infusion once monthly (1 course = 1 month). The dosage was 12 mg/kg for patients <30 kg and 8 mg/kg for those ≥30 kg. Chest high-resolution computed tomography (HRCT) scans to evaluate pulmonary involvement were routinely repeated every 6 months.

Pt, patient; TCZ, tocilizumab; GCs, glucocorticoids; MTX, methotrexate; MMF, mycophenolate mofetil; CYC, cyclophosphamide; DLCO, diffusing capacity for carbon monoxide (% predicted); FVC, forced vital capacity (% predicted); mRSS, modified Rodnan skin score; J4s, Juvenile Systemic Sclerosis Severity Score; NA, not available.

CT changes: +, abnormal; −, normal; + → −, data not available.

*Patient 11 discontinued TCZ due to leukopenia and was excluded from the pooled statistical analysis. The pooled row shows the median (range) for # of courses and follow-up duration, and the mean ± SD for the mRSS and J4s. p = 0.028 for mRSS (Wilcoxon), p = 0.004 for J4s (paired t-test).

Pooled analysis of 10 evaluable patients (excluding one patient who discontinued treatment due to leukopenia) showed that the median number of tocilizumab courses was 16 (range 3–30). The pre- and posttreatment mRSS decreased from 9.6 ± 8.7 to 5.4 ± 5.5 (43.8% reduction, p = 0.028, Wilcoxon signed-rank test), and the J4s decreased from 5.7 ± 1.4 to 2.5 ± 1.5 (56.1% reduction, p = 0.004, paired t-test). It should be noted that all patients concurrently received GCs and/or DMARDs.

## Discussion

As a rare disease, jSSc has limited clinical research. Our center11-year single-center, retrospective analysis preliminarily revealed the clinical characteristics of the current diagnosis, objective evaluation index, and treatment status of jSSc in China. In this study, female patients accounted for 71.4%, consistent with the female predominance reported in previous international studies (4–7, 20). This suggests that gender may play an important role in jSSc pathogenesis, presumably related to estrogen’s regulatory effects on the immune system.

The diffuse-type proportion in this study was 66.7%, approaching the approximately 70% reported in international studies. A Korean 31-year cohort study reported that 65.4% (13 cases) of patients had the diffuse type (6), while a French cohort showed 18 cases, accounting for only 44%, suggesting potential subtype distribution differences across different regions and ethnicities (4). Regarding clinical manifestations, skin involvement was a universal symptom in all patients (100%), while Raynaud’s phenomenon incidence was only 19.0%, lower than the 50%–80% reported in most previous studies (4, 6), which could be attributed to insufficient recognition of Raynaud’s phenomenon by patients and families during history collection, potential racial variations in the clinical phenotype of Chinese jSSc, or the presence of atypical symptoms in early-stage cases. Since Raynaud’s phenomenon is an early common manifestation of jSSc and guidelines recommend early nailfold capillaroscopy and ANA testing for children to screen jSSc, clinicians need to enhance recognition of Raynaud’s phenomenon. Especially for children with unexplained skin sclerosis and joint symptoms, relevant medical history should be actively sought to avoid misdiagnosis and diagnostic delays (21).

Organ involvement in jSSc patients in this study was mainly concentrated in the lungs (28.6%) and gastrointestinal tract (23.8%), with a cardiac involvement rate of only 4.8% and no renal crises. This is consistent with commonly reported pulmonary involvement and rare renal crises in some international studies. However, some studies reported higher cardiac involvement rates in the limited type, possibly related to differences in sample subtype distribution, follow-up duration, and detection methods (16). Specifically, a recent multicenter study emphasized that cardiac manifestations are often subclinical and require systematic longitudinal monitoring, as detection rates vary significantly based on whether cardiac MRI or advanced echocardiography is utilized (22). It should be noted that with a median follow-up time of 2.3 years (range 0.5–7.2 years) in this study, the conclusion of no deaths or renal crises is limited by the relatively short follow-up duration and cannot represent long-term prognosis. As one of the major causes of death in jSSc, three cases in this study, combined with interstitial lung disease, showed stable conditions after active treatment, suggesting that early intervention can effectively prevent pulmonary disease progression.

Regarding autoantibody testing, the ANA positivity rate in this study was 76.2%, with an anti-Scl-70 antibody positivity rate of only 9.5%, far lower than that of adult systemic sclerosis (approximately 30%–40%). A Japanese nationwide jSSc survey showed an anti-Scl-70 positivity rate as high as 62.4%, and the ACR conference-reported jSSc cohort showed an anti-Scl-70 positivity rate of 14.3% (23). This difference may reflect variations in autoantibody profiles between jSSc and adult SSc and across different ethnicities. Asian adult SSc patients typically have higher anti-Scl-70 positivity rates than Caucasian patients (30%–80% vs 14%–35%) and are associated with more severe clinical phenotypes (2, 24). Our cohort showed distinctly different antibody profile characteristics: relatively high anti-ssDNA and anti-PM-Scl antibody positivity rates, with extremely low anti-Scl-70 positivity rates. This unique antibody profile feature may suggest different immunological mechanisms between pediatric and adult SSc. Five patients were completely negative for autoantibodies (23.8%), suggesting that some jSSc patients may lack typical autoantibodies; clinical diagnosis should comprehensively consider clinical manifestations and imaging examinations to avoid missed diagnosis due to overreliance on antibody testing.

GCs and methotrexate remain the cornerstone therapeutic drugs in this study. For children with poor traditional treatment outcomes, biologic agents show good application prospects. Tocilizumab is a humanized monoclonal antibody that competitively inhibits IL-6 binding to both soluble and membrane-bound receptors. In jSSc, IL-6 acts as a critical link between persistent inflammation and progressive fibrosis by promoting myofibroblast differentiation (25, 26). The role of tocilizumab in jSSc differs significantly from other pediatric rheumatic diseases: while in juvenile idiopathic arthritis (JIA), it primarily targets systemic inflammatory cytokines and synovial pannus, and in juvenile dermatomyositis (JDM), it addresses refractory muscle inflammation. Its utility in jSSc is focused on interrupting the IL-6-driven fibrotic cascade to improve skin and lung compliance (26). Eleven patients receiving tocilizumab treatment in this study showed varying degrees of improvement in skin sclerosis, joint symptoms, and pulmonary function, consistent with the study results of Adrovic et al. (27).

However, caution must be exercised when evaluating tocilizumab efficacy. First, all patients receiving tocilizumab treatment in this study concurrently used GCs and/or traditional DMARDs (such as methotrexate and mycophenolate mofetil), and the lack of a control group prevents us from distinguishing whether improvements came from tocilizumab or baseline immunosuppressive therapy. The focal trial in adult SSc is a randomized controlled study of tocilizumab treatment for SSc (28). Although this study did not meet primary skin endpoints, it showed that FVC decline in the tocilizumab group was slower than that in the placebo group, suggesting potential protective effects on pulmonary involvement. Therefore, we believe that tocilizumab as part of combination therapy demonstrates good safety and potential adjunctive efficacy, but its independent efficacy still requires confirmation through larger-scale, well-designed controlled studies. Rituximab is an anti-CD20 monoclonal antibody that can deplete B lymphocytes and inhibit autoantibody production (29). One patient in our study showed symptom improvement after rituximab use, consistent with findings by Zulian et al. (30). Additionally, JAK inhibitors such as tofacitinib have shown treatment potential for refractory jSSc in case reports (31), but their application in children still requires more clinical data support.

This study significantly contributes to the literature by providing one of the few longitudinal analyses of an 11-year Chinese jSSc cohort. Our findings highlight a distinct autoantibody profile (predominantly anti-ssDNA and anti-PM-Scl) that differs from that of previously published Western and Japanese cohorts, suggesting ethnic-specific immunological phenotypes. Furthermore, our real-world application of the J4s and mRSS scores demonstrates their value as dynamic tools for a “treat-to-target” strategy in pediatric clinical practice, providing a benchmark for monitoring multiorgan outcomes in Asian patients.

Our study has several limitations inherent to its design. As a single-center, retrospective analysis with a modest sample size, the median follow-up duration of 2.3 years is relatively short, and the observation of no mortality or scleroderma renal crisis should be interpreted with caution. Additionally, the absence of a control group and the universal use of concomitant background immunosuppressive therapy preclude definitive attribution of observed clinical improvements to tocilizumab alone. Meanwhile, the completeness of some laboratory and imaging data was insufficient. Future prospective, multicenter studies with larger cohorts are warranted to better characterize the clinical phenotype and pathogenesis of juvenile systemic sclerosis in jSSc and to optimize therapeutic strategies. Establishing longitudinal registries will also be crucial for monitoring long-term prognosis and treatment safety.

## Conclusions

The early manifestations of jSSc may be atypical, and the diagnosis can be complex. Timely recognition and early intervention are essential to improve outcomes. The J4s score and mRSS can serve as reliable, objective assessment tools for monitoring disease severity and guiding “treat-to-target” therapeutic decisions. Biologic agents such as tocilizumab have demonstrated safety and potential adjunctive efficacy within combination regimens by targeting IL-6-mediated fibrotic pathways. Future efforts should prioritize prospective, multicenter studies with extended follow-up to refine diagnostic and therapeutic approaches for jSSc.

## Data Availability

The original contributions presented in the study are included in the article/supplementary material. Further inquiries can be directed to the corresponding authors.
